# Studying fitness cost of *Plasmodium falciparum* infection in malaria vectors: validation of an appropriate negative control

**DOI:** 10.1186/1475-2875-12-2

**Published:** 2013-01-02

**Authors:** Ibrahim Sangare, Yannis Michalakis, Bienvenue Yameogo, Roch Dabire, Isabelle Morlais, Anna Cohuet

**Affiliations:** 1 Institut de Recherche en Sciences de la Santé-Direction Régionale de l’Ouest, Bobo Dioulasso, Burkina Faso; 2 Institut de Recherche pour le Développement, Unité MIVEGEC (IRD 224-CNRS 5290-UM1-UM2), BP 64501, Montpellier Cedex 5, 34394, France; 3 Laboratoire de Recherche sur le Paludisme, Organisation de Coordination pour la lutte contre les Endémies en Afrique Centrale, Yaoundé, BP 288, Cameroon

**Keywords:** *Plasmodium falciparum*, Vectors, Infection cost, Negative control, Fitness

## Abstract

**Background:**

The question whether *Plasmodium falciparum* infection affects the fitness of mosquito vectors remains open. A hurdle for resolving this question is the lack of appropriate control, non-infected mosquitoes that can be compared to the infected ones. It was shown recently that heating *P. falciparum* gametocyte-infected blood before feeding by malaria vectors inhibits the infection. Therefore, the same source of gametocyte-infected blood could be divided in two parts, one heated, serving as the control, the other unheated, allowing the comparison of infected and uninfected mosquitoes which fed on exactly the same blood otherwise. However, before using this method for characterizing the cost of infection to mosquitoes, it is necessary to establish whether feeding on previously heated blood affects the survival and fecundity of mosquito females.

**Methods:**

*Anopheles gambiae* M molecular form females were exposed to heated *versus* non-heated, parasite-free human blood to mimic blood meal on non-infectious *versus* infectious gametocyte-containing blood. Life history traits of mosquito females fed on blood that was heat-treated or not were then compared.

**Results:**

The results reveal that heat treatment of the blood did not affect the survival and fecundity of mosquito females. Consistently, blood heat treatment did not affect the quantity of blood ingested.

**Conclusions:**

The study indicates that heat inactivation of gametocyte-infected blood will only inhibit mosquito infection and that this method is suitable for quantifying the fitness cost incurred by mosquitoes upon infection by *P. falciparum.*

## Background

Mosquito fitness is a crucial factor of vectorial capacity and malaria transmission [1]. However, the question whether *Plasmodium* parasites affect the fitness of mosquito vectors they infect remains open. Ten years ago, a review pointed out conflicting results in previous studies according to the design of experiments, environmental conditions, and the parasite-mosquito species used [2]. This underlined the need to develop experiments using natural vector-parasite species combinations in controlled environmental conditions. In the last decade, additional studies aimed at deciphering the cost of infection in malaria vectors by using different species and methods and produced again contrasted results [3-7]. Some studies showed a shorter lifespan in infected mosquitoes or no detected effect of infection depending on the parasite load and/or environmental conditions [3,5-7]. Recently, another study observed a longer lifespan in infected mosquitoes associated with a lower fecundity [4]. Concerning the most important vectorial system for human malaria: *Anopheles gambiae/Plasmodium falciparum*, some studies suggested an infection cost in this combination of species [7,8], others did not find it [9,10]. Further investigations are therefore needed to determine how infection affects the mosquito vector in this epidemiologically relevant system.

Measuring how parasite infection affects fitness traits requires the comparison of infected and non-infected mosquitoes, either collected from the field or produced in laboratory conditions. Comparison of field-collected infected and non-infected mosquitoes is limited by the difficulties to obtain a large number of infected mosquitoes [7], and by the lack of a reliable method for ageing the field-collected mosquitoes [8]. In the laboratory, obtaining a large number of *Plasmodium*-infected mosquitoes is facilitated by exposing insectary-reared *Anopheles* to gametocytes through membrane feeding (gametocyte-infected blood from naturally infected patients in direct membrane feeding assays, DMFA, or gametocyte-containing parasite culture in standard membrane feeding assays, SMFA) [11]. Different methods were used so far for producing non-infected mosquitoes to be compared to the ones exposed to *Plasmodium* gametocytes. In DMFA, in parallel to the experimental infection of mosquitoes, other females were exposed to parasite-free blood from another volunteer [10,12]; in SMFA the control mosquitoes were fed on a parasite culture not producing gametocytes [13]. Also exposing the mosquitoes that ingested infectious gametocytes to high temperatures just after the blood feeding can limit the infection success and produce non-infected mosquitoes [13]. All these methods however, incur the problem of confounding factors: when using the blood of uninfected hosts, one cannot rule out that host effects are responsible for the potential differences in mosquito life-history traits [14]; also, parasite strain-specific effects, or temperature effects [15,16] may be confounded with the effect of infection. This underlines a need for a proper control for evaluating the cost of infection.

It was shown recently that heating *P. falciparum* gametocyte-infected blood before feeding by malaria vectors impedes the infection by killing or inhibiting the infectivity of gametocytes [17,18]. This potentially allows the comparison of infected to non-infected mosquitoes that fed on the same blood at the same time. However, before using this method for characterizing the cost of infection to mosquitoes, it is necessary to establish whether feeding on previously heated blood affects the survival and fecundity of mosquito females. Therefore, life history traits of female mosquitoes exposed to non-infected blood that was subjected or not to a heat treatment, were compared. This allowed validating the method of using heat inactivation of *P. falciparum* gametocytes for comparison of fitness traits between infected and non-infected *An. gambiae*.

## Methods

### Mosquitoes

A mosquito colony of *An. gambiae* M molecular form was used. The colony was established in 2008 from wild-caught gravid females collected in Kou Valley (30 km north from Bobo Dioulasso), Burkina Faso [19]. The females laid eggs individually before their species was determined by PCR-RFLP [20] and M form egg batches were pooled. The colony was maintained in the insectary under standard conditions (12 h day/night cycle, 28+/−2°C, 80+/−5% humidity).

The first blood meal of *An. gambiae* females is potentially used to compensate for nutritional deficiencies carried over from larval life and induce complete egg development and laying only in bigger females, whereas almost all females are able to fully develop eggs at subsequent blood meals [21-23]. The second blood meal is therefore more representative of female fecundity along its lifespan and egg production was observed after the second blood meal. Before measures of life history traits, females were fed on rabbit blood three days after adult emergence and on human blood the fifth or sixth day of adult life. Sugar was removed 36 h prior to blood feeding. For mosquito feeding on human blood, venous blood was collected from five volunteers, non-infected by *P. falciparum*, who were enrolled after written informed consent. Ethical approval was obtained from the relevant institutional Ethics Committee.

### Blood feeding

Non-infected human blood was treated in the same manner as for experimental infections of malaria vectors by DMFA using natural isolates of *P. falciparum* and using gametocyte heat inactivation in parallel [17,18]. The blood was first centrifuged at 2,000 rpm at 37°C for three min, and the serum was replaced by the same volume of European AB serum. In experimental infections, this step limits the effect of human transmission blocking immunity [24]. To mimic gametocyte heat inactivation, half of the reconstituted blood was placed in a thermo-mixer and heated at 43°C for 15 min and 900 rpm while the remaining blood was maintained at 37°C. Five hundred μl of blood (heated or not) were distributed in membrane feeders maintained at 37°C by water jackets. At least two different feeders were used for each group (blood donor and heat treatment) in order to limit potential feeder effects. Cups containing 50 mosquito females were placed under the feeders to allow blood feeding through Parafilm membranes for 30 min. Fed females were sorted and placed in individual 30 ml plastic tubes for subsequent measures of life history traits. Because of logistic limitations, the blood feeding with the different blood donors had to be carried out at different days and using different mosquito batches from the same colony and therefore constituted different experimental blocks. In the statistical analyses the effect “blood donor” could thus be due to either differences in the “quality” of the blood of the various donors, intrinsic differences of the different mosquito batches, date effects, or a combination of the above.

### Measure of life history traits

From the day of membrane blood feeding on heated *versus* non-heated blood and until death of all mosquitoes, females were observed every eight hours. Dead females were immediately processed: ovaries were dissected for egg counting and wings were mounted on slides to measure their length. If both wings of a female were intact one wing was randomly chosen to be measured. When one wing was damaged, the undamaged wing was measured. Wing size was measured from the alula to the wing tip, excluding scales as previously described [25] by using the software ImageJ [26].

The amount of haematin, a by-product of the decomposition of haemoglobin, was measured for each female [27] to obtain a relative measure of blood meal size. For all mosquitoes that died at least three days after blood feeding and therefore completed digestion and excreted all the haematin, 1 ml of 1% LiCO_3 _was distributed in individual tubes to elute faeces. The absorbance of the resulting solution was read at 387 nm, using LiCO_3 _solution as a blank, and compared with a standard curve made with porcine serum haematin (Sigma-Aldrich).

### Statistical analyses

To investigate whether the blood heating treatment affects mosquito survival, Kaplan-Meier survival estimates were calculated and the effects of heat treatment, blood donor, and their interaction, were tested using Cox's Proportional Hazards model. Wing length and the quantity of excreted haematin were used as co-variables.

Preliminary analysis of the results on fecundity revealed that a variable proportion of females did not develop any eggs, thus yielding distributions with a spike at zero and a more or less normal distribution for larger values of the number of eggs produced. The analysis of fecundity results were thus split in two parts. First, nominal logistic regressions were used to test whether heat treatment affects the probability that a female may develop at least one egg. Because the size of an individual and its blood meal size may affect female fecundity wing length and quantity of haematin excreted were included as co-variables in the model. Second, the effect of blood treatment on the number of eggs developed per female, exclusively on females that developed at least one egg, was tested with an analysis of variance including wing length and quantity of haematin excreted as co-variables.

In order to investigate whether heating the blood may influence the quantity ingested by a female, the potential effect of the heat treatment on the blood meal size was tested with an analysis of variance with blood donor as a co-variable.

In the analyses, the blood-donor effect is considered as fixed. The choice of this option was done because (i) fixed effects models are easier to present than mixed effects models; (ii) when declaring the blood-donor effect and its interaction with the heat treatment as random effects, similar conclusions were always reached; (iii) the small number of blood donors (five) legitimately questions whether they can be considered as a representative sample of the blood donor population.

## Results

### Survival

**Figure 1:**
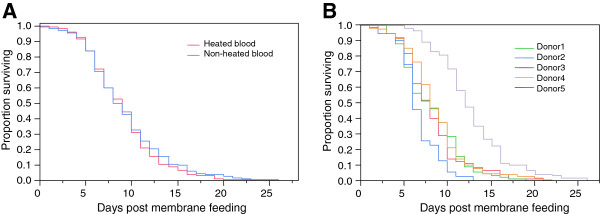
**Survival curves.** Kaplan-Meier estimates of survival of female mosquitoes after a blood meal as a function of whether the blood had been previously heated (in red) or not (in blue) (**A**) or as a function of blood donor (**B**). In A, survival curves between females fed on heated versus non-heated blood are not significantly different according to the Cox Proportional Hazards tests (P = 0.46). In B, survival curves between females fed on blood from different blood donors are significantly different according to the Cox Proportional Hazards tests (P <0.001).

A total of 485 *An. gambiae* females were included in the analysis, ranging from 70 to 131 per blood donor. The Cox Proportional Hazards model revealed that the blood heating treatment, whether alone or in interaction with the blood donor, did not affect mosquito survival (Table 1, Figure 1A). In contrast, a significant difference in survival among females that fed on blood from different donors was evidenced (Table 1, Figure 1B). Similar conclusions were obtained if instead of carrying out the analysis on survival after the membrane blood meal the total adult lifespan since emergence was used (results not shown). Longevity was highly different between mosquito batches fed on different blood donors, with median day of death post membrane feeding ranging from six (blood donor 2) to 12 (blood donor 3), corresponding to 11 and 17 days post-emergence respectively. Longevity was also significantly affected by wing length and the quantity of haematin excreted (Table 1). The estimates indicated that the former relation is negative and the latter positive (not shown). This suggests a longer lifespan for the females that ingested more blood, while larger females died faster.

**Table 1:** **Statistical analysis of effects on *Anopheles gambiae *females survival**

**Source**	**DF**	***X***^**2**^	***P value***
Heat treatment	1	1.441	0.2300
Blood donor	4	95.705	<.0001
Heat treatment* Blood donor	4	3.114	0.5390
Wing length	1	8.087	0.0045
Haematin	1	10.240	0.0014

Analysis of the effect of blood heat treatment, blood donor and their interaction on female mosquito survival after the blood meal using the Cox Proportional Hazards model.

### Fecundity

**Figure 2:**
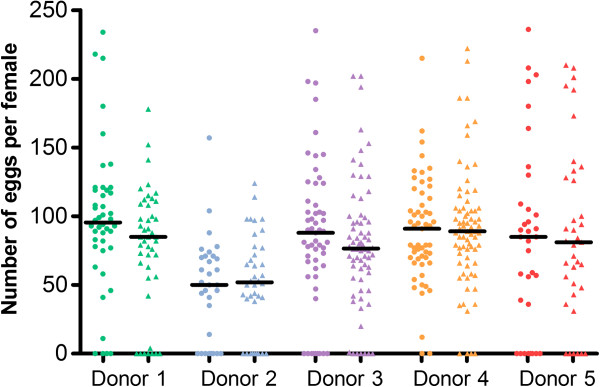
**Eggs numbers.** Number of eggs per dissected female for each blood donor and blood treatment (circles for heated blood and triangles for non-heated blood). Horizontal bars indicate median.

The proportion of females that did not develop any egg varied significantly among blood donors and was negatively influenced by blood meal size: females that feed more blood were more prone to develop at least one egg. In contrast, the proportion of females that did not develop any egg was not affected by the blood treatment or female wing length (Table 2). For females that developed at least one egg, blood treatment did not affect the number of eggs developed by each female. There was significant variation among donors, the average number of eggs per female across patients ranging from 66 to 107 (Table 3, Figure 2). The number of eggs was also significantly larger in bigger females and females that ingested larger blood meals (Table 3).

**Table 2:** **Statistical analysis of effects on development of at least one egg in *Anopheles gambiae ***

**Source**	**DF**	***X***^**2**^	***P value***
Heat treatment	1	0.304	0.5816
Blood donor	4	5.611	0.2302
Heat treatment *Blood donor	4	1.829	0.7672
Wing length	1	1.811	0.1784
Haematin	1	20.151	<.0001

Nominal logistic regression on whether a female laid any eggs as a function of blood treatment, blood donor, their interaction, female wing length and quantity of haematin excreted.

**Table 3:** **Statistical analysis of effects on number of eggs developed in*Anopheles gambiae***

**Source**	**DF**	**Sum of squares**	**F ratio**	**Prob > F**
Heat treatment	1	1921.585	1.3775	0.2414
Blood donor	4	24304.107	4.3557	0.0019
Heat treatment *Blood donor	4	8354.035	1.4972	0.2027
Wing length	1	68160.616	48.8617	<.0001
Haematin	1	3889.278	2.7881	0.0959

Analysis of variance of the number of eggs developed per female as a function of blood treatment, blood donor and their interaction, female wing length and haematin excreted (model R^2^ = 0.24).

### Blood meal size

The analysis did not detect any effect of blood heat treatment on the quantity of blood ingested by female mosquitoes. However, the donor effect was significant (Table 4), suggesting that the blood meal size taken by the mosquitoes differs between blood donor.

**Table 4:** **Statistical analysis of effects on blood meal size in *Anopheles gambiae ***

**Source**	**DF**	**Sum of squares**	**F ratio**	**Prob > F**
Heat treatment	1	3.4092	0.0756	0.7835
Blood donor	4	1165.2930	6.4596	<.0001
Heat treatment *Blood donor	4	346.0512	1.9183	0.1062

Analysis of variance of the quantity of haematin excreted as a function of blood treatment and blood donor (model R^2^ = 0.07).

## Discussion

In the present study, it was investigated whether a heat treatment of human blood that mimics heat inactivation of *P. falciparum* gametocytes affects the fitness of the vector mosquitoes. Life history traits of *An. gambiae* females fed on parasite-free blood that was heat treated to that of females fed on not-treated blood were compared. The results revealed that heat treatment of the blood did not affect the survival and fecundity of mosquito females. Consistently, blood heat treatment did not affect the quantity of blood ingested. Then, it can be conjectured that heat inactivation of gametocyte-infected blood will only inhibit mosquito infection and that this method is suitable for quantifying the fitness cost incurred by mosquitoes upon an infection by *P. falciparum*.

Mosquito body size can affect both longevity and fecundity e.g. [21,28-30], either directly or indirectly through its potential effect on the quantity of ingested blood [31,32]. These variables were therefore included into the analysis. The results evidenced positive relations of both the body size, estimated by wing length, and the blood meal size, estimated from the quantity of haematin excreted, with the number of eggs developed. This confirms that bigger females and the ones that ingest more blood have higher fecundity, as previously observed in *An. gambiae* and other mosquito species [33-35]. Blood meal size was also found positively related to longevity but bigger females were found to die faster. This result indicates that nutritive resources of the blood meal are used by the mosquito females for survival; however there is no clear explanation for why larger females died faster in this experiment. A trade-off between fecundity and longevity could explain this relation, but the data did not support this hypothesis.

The present study revealed large variations in fecundity and longevity of mosquitoes between replicates. The replicates differed by the batch of mosquitoes, taken from same mosquito colony, the day of experiment, and the blood donor. It is therefore not possible to conclude whether differences are due to blood characteristics or uncontrolled variations in insectary conditions that affect mosquito fitness. Further experiments will be needed to determine the effect of human blood variations on mosquito fitness, which could be a key parameter of mosquito preference for blood feeding and then malaria transmission [36,37].

## Conclusion

This study aimed at validating an accurate method for producing control non-infected mosquitoes to be compared with *P. falciparum*-infected mosquitoes for measures of life history traits. The blood heat treatment at 43°C for 15 min and 900 rpm, which was previously demonstrated to effectively hinder *P. falciparum* infectivity, does not affect mosquito longevity and fecundity. Therefore, this method can be used to feed simultaneously mosquitoes on gametocyte-containing blood *versus* heat-inactivated blood from the same donor. This procedure will enable accurate characterization of the cost of *P. falciparum* infection incurred by mosquitoes while avoiding potentially confounding factors from parasites or blood donors.

## Competing interests

The authors declare that they have no competing interests.

## Authors’ contributions

IS, BY carried out the experiments, IS drafted the manuscript. YM participated in the design of the study and carried out the statistical analysis. RD and IM participated in the design of the study. AC conceived of the study, and participated in its design and coordination. All authors read and approved the final manuscript.
